# A Pathway Analysis Based on Genome-Wide DNA Methylation of Chinese Patients with Graves' Orbitopathy

**DOI:** 10.1155/2019/9565794

**Published:** 2019-01-13

**Authors:** Zhong Xin, Lin Hua, Yi-Lin Yang, Ting-Ting Shi, Wei Liu, Xiu Tuo, Yu Li, Xi Cao, Fang-Yuan Yang

**Affiliations:** ^1^Department of Endocrinology, Beijing Tongren Hospital, Capital Medical University, Beijing, China; ^2^Department of Mathematics, School of Biomedical Engineering, Capital Medical University, Beijing, China; ^3^Physical Examination Department, Beijing Tongren Hospital, Capital Medical University, Beijing, China

## Abstract

**Background:**

The pathogenesis Graves' Orbitopathy (GO) is not yet fully understood. Here, we conducted a pathway analysis based on genome-wide DNA methylation data of Chinese GO patients to explore GO-related pathways and potential feature genes.

**Methods:**

Six GO patients and 6 age-matched control individuals were recruited, and a genome-scale screen of DNA methylation was measured using their peripheral blood sample. After extracting the differentially methylated regions (DMRs), we classified DMRs into three clusters with respect to median absolute deviation (MAD) for GO and control group, respectively. Then the extract tests were performed to identify significant pathways by comparing the counts of genes in each cluster between GO and control group in a pathway. For each significant pathway, we calculated the Methylation-based Inference of Regulatory Activity (MIRA) score to infer the regulatory activity of genes involved in the pathway. Furthermore, we took the significant pathways as the subsets and applied Random forests (RF) method to extract GO-related feature genes.

**Results:**

We identified four potential significant pathways associated with the occurrence and development of GO disease. There were Toxoplasmosis, Axon guidance, Focal adhesion, and Proteoglycans in cancer (p<0.001 or p=0.007). The identified genes involved in the significant pathways, such as LDLR (p=0.019), CDK5 (p=0.036), and PIK3CB (p=0.020), were found to be correlated with GO phenotype.

**Conclusion:**

Our study suggested pathway analyses can help understand the potential relationships between the DNA methylation level of some certain genes and their regulation in Chinese GO patients.

## 1. Introduction

Graves' Orbitopathy (GO), an autoimmune disease that is associated with a wide spectrum of ocular changes, is a difficult challenge in endocrinology and ophthalmology. GO often occurs in patients with abnormal thyroid function, such as hyperthyroidism. The clinical manifestations include extraocular muscle enlargement and orbital fat expansion [1]. Because the pathogenesis of GO involves complex molecular and cellular processes that have not yet been fully clarified [2], the present treatments do not target its pathogenic mechanisms [3].

DNA methylation (DNAm) affects gene expression, cellular differentiation, and molecular response to environmental factors. DNAm patterns changes may explain the increased risk of some diseases. In our previous study, we found that DNAm differences were associated with GO patients from a genome-wide DNA methylation analysis in peripheral blood. Several genomic loci were identified with significant differences in methylation patterns that were associated with GO incidence [4]. ClusterProfiler tool [5] was used to perform Gene Ontology and Kyoto Encyclopedia of Genes and Genomes (KEGG) pathway enrichment analysis for genes near or at the differentially methylated DNA regions. However, we did not find any significant enriched pathways in KEGG pathway with the enrichment analysis.

The disease heterogeneity and complexity indicate that GO is not caused by any single gene but caused by the complex regulation among multiple genes [6]. Currently, the identification of the genes associated with complex GO disease has become an important task to help find the pathology of this disease. It is known that studies on analyzing gene function have provided valuable insights into the functional properties of gene groups [7]. Meanwhile, by combining a priori available biological knowledge, pathway analyses have emerged as important tools to uncover functional networks of genome-wide data [8]. Thus, pathway analyses are important to study molecular metabolize mechanism of GO disease, and the identification of disease-related pathways can help find disease-related feature genes which are related to particular pathways.

In our previous study, the general enrichment analysis based on hypergeometric distribution test did not find any significant pathway, which might be due to the following two reasons: (1) the multiple tests corrections eliminate the positive results; (2) the analysis is based on gene lists defined by a cut-off and does not take into account the expression distribution of genes in the pathway. Indeed, methods for investigating how changes in gene expression variability in the context of pathways can help find pathways and the gene function sets. Here, we reanalyzed our previous data and adopted a novel pathway analysis to extract significant pathways. This method can identify pathways associated with different patterns of expression variability which may highlight those pathways that contribute to group-specific differences [9]. In the present study, the differentially methylated regions (DMRs) were reclassified into different clusters according to median absolute deviation (MAD) for GO and control group, respectively. The extract tests were performed to extract significant pathways based on the comparison of the counts of genes in each cluster in a pathway. The results of this study may provide new insights for better understanding the pathophysiologic mechanism of GO.

## 2. Materials and Methods

### 2.1. Study Subjects

DNA was obtained from 6 Chinese patients with GO as well as 6 age-matched controls who had normal thyroid function and no clinical manifestations of GO. The diagnosis of GO was based on the EUGOGO consensus [10]. The seven-point Clinical Activity Score (CAS) was recorded by using the modified EUGOGO patient form. TRAb was measured using commercially available electrochemiluminescence assays based on the M22 monoclonal antibody, with a normal range <1.75 U/L (Roche Diagnostics GmbH). None of the patients received any immunosuppressive therapy or radiotherapy previously (Table 1). The study was conducted with the approval from the Ethics Committee of Beijing Tongren Hospital, Capital Medical University. Written informed consent was obtained from each participant [4].

### 2.2. RRBS

In the present study, RRBS (Reduced Representation Bisulfite Sequencing) assay was performed [11]. DNA was purified using AMPure XP beads and the A-tailed DNA fragments were subsequently ligated with methylated-adapters. The purified size-selected DNA fragments were bisulfite converted and subsequently amplified by PCR. PCR products were then purified using AMPure XP beads, and PCR amplified RRBS libraries were then quantified. Finally, 10G of 2x150 bp pair-end raw data was generated, each sample on the Illumina Hiseq 2500 platform. The Adapter-trimmed and quality-filtered clean reads were aligned to the bisulfite converted reference genome hg19 and the methylation level of cytosine in CpG, CHG and CHH context were calculated separately, through which the methylation levels of CPGI, gene and Transcription start sites (TSS) regions were also calculated. The detailed description was seen from our previous study [4].

### 2.3. Extraction of Significant Pathways

Firstly, we used methylKit [12] and eDMR [13] software to extract differentially methylated regions (DMRs) in genomic regions. P<0.05 is considered as significant. According to this criterion, 841 differentially DMRs were identified [4]. It is known that the pathway analyses are important to study molecular metabolize mechanism of disease; we thus applied a novel analysis method to investigate changes in gene expression variability in the context of gene subsets to extract significant pathways [9]. In this method, genes were classified into one to four clusters with respect to the variability statistics such as standard deviation (SD), median absolute deviation (MAD), and coefficient of variation (CV). In theory, it would like to choose the variability statistics that has the smallest correlation with the mean. After performing the correlation analysis, we found that MAD has the least correlation with the mean, and we therefore implement further analysis based on it. With respect to the MAD, we classified DMRs (genes) into three clusters for GO and control group respectively. Then the extract tests were performed to extract significant pathways based on the comparison of the counts of genes in each cluster in a pathway between GO and control group. Those pathways with P<0.05 were considered as significant. We used pathVar package of R software (http://www.r-project.org) to implement this analysis.

### 2.4. Calculation of MIRA Score for Genes Involved in the Significant Pathways

The concept of the Methylation-based Inference of Regulatory Activity (MIRA) score [14] relies on the observation that DNA methylation tends to be lower in regions where transcription factors are bound. Since DNA methylation will generally be lower in active regions, the shape of the MIRA profile and the associated score can be used as a metric to compare regulatory activity in different samples and conditions. MIRA overcomes sparsely in DNA methylation data by aggregating across many regions and thus can help infer the regulatory activity from DNA methylation data. By aggregating DNA methylation data from a set of regions across the genome, the algorithm produces a single summary profile of DNA methylation for those regions. Then this profile is used to produce MIRA score which infers the level of regulatory activity on the basis of the shape of the DNA methylation profile. MIRA can also work with genome-scale RRBS data.

Here, for each of significant pathways, we calculated the MIRA score of each sample to infer the regulatory activity of genes involved in this pathway. We used MIRA package of R software (http://www.r-project.org) to implement the analysis. In addition, for each significant pathway, we applied the independent two sample t test to compare MIRA scores between GO and control group.

### 2.5. Random Forest Analysis to Extract GO-Related Feature Genes Involved in the Significant Pathways

**Figure 1:**
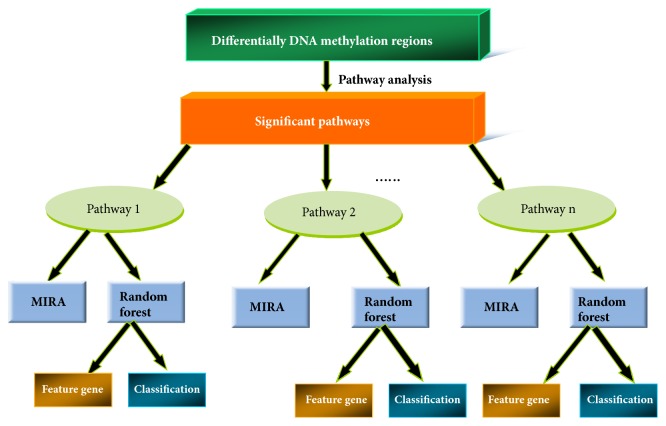
The flowchart of the study. Firstly, the pathway analyses were performed to extract the significant pathways based on the DNA methylation profile of the differentially methylated regions (DMRs). Secondly, for each significant pathway, the Methylation-based Inference of Regulatory Activity (MIRA) scores were calculated to infer the regulatory activity of genes involved in the pathway. Finally, the genes involved in the significant pathways were taken as the subsets, and random forest method was applied to these gene subsets to classify samples and to extract GO-related feature genes. DMRs: differentially methylated regions. MIRA: Methylation-based Inference of Regulatory Activity.

Random forests (RF) method is an ensemble classifier that consists of many decision trees and each tree depends on the values of a random vector sampled independently [15]. As a brief description, RF method selects a random sample of observation and randomly takes initial variables to build decision tree model. This process is repeated until getting a final prediction which is a function of each prediction on each observation. Here, for each significant pathway, we applied RF method to distinguish GO from control group based on the DNA methylation data of genes involved in this pathway. Notably, a prediction model of machine learning algorithms is usually constructed based on the training dataset and is evaluated using the testing dataset. In order to avoid the problems like overfitting, we adopt leave-one-out (LOO) to implement the analysis. That is, for each analysis, one sample was considered as testing data whereas the remaining samples were considered as training data to construct random forest model. Then each significant pathway was taken as the subset and RF method was applied to extract GO-related feature genes involved in this pathway. The RF method to obtain feature gene G from an important pathway is permuting the DNA methylation value of gene G of out of bag according to random forest model. If gene G is a good predictor, then it will appear in a large number of split trees. Here, we used Mean Decrease Gini (MDG) to evaluate whether gene G is a feature gene or not. MDG was the total decrease in node impurities measured by the Gini index from splitting on the variable, averaged over all trees. It provided possible ways to quantify which genes contribute most to classification accuracy. Greater MDG will indicate that the degree of impurity arising from category could be reduced farthest by gene G and thus suggests an important feature gene. Because we adopt LOO method in the current study, thus 12 random forest models were constructed for each significant pathway. In each pathway, we extracted those genes whose MDG ranked the top 5 in all of 12 models as GO-related feature genes. RF method was implemented with the randomForest package of R software (http://www.r-project.org). The flowchart of our work was shown in Figure 1.

## 3. Results

### 3.1. Identification of Differentially DNA Methylation Regions

According to the criterion of p < 0.05, 1583 differentially DNA methylation regions were identified. After removing those regions obtained from missing samples, 841 differentially methylated regions were extracted [4] and were used for further analysis.

### 3.2. Extraction of Significant Pathways

**Figure 2:**
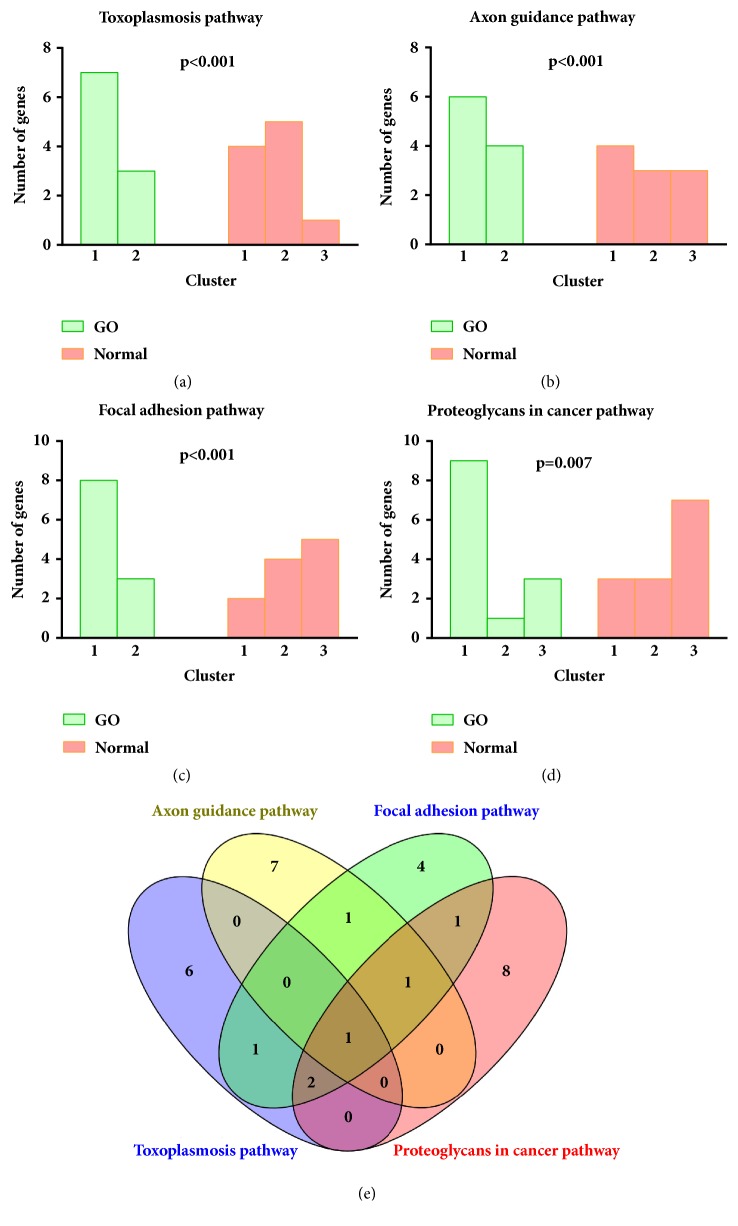
Four significant pathways with GO. (a) The distribution of the counts of genes in three clusters in toxoplasmosis pathway between GO patients and normal controls. (b) The distribution of the counts of genes in three clusters in Axon guidance pathway between GO patients and normal controls. (c) The distribution of the counts of genes in three clusters in focal adhesion pathway between GO patients and normal controls. (d) The distribution of the counts of genes in three clusters in Proteoglycans in cancer pathway between GO patients and normal controls. (e) A Venn diagram to show the number of overlapped genes shared by four significant pathways.

In order to extract significant pathways, we classified DMRs into three clusters with respect to MAD for GO and control group, respectively. Then the extract tests were performed to extract significant pathways based on the comparison of the counts of genes in each cluster in a pathway between GO and control group. According to p<0.05, four significant pathways were extracted (Table 2 and Figures 2(a), 2(b), 2(c), and 2(d)). There were Toxoplasmosis, Axon guidance, Focal adhesion, and Proteoglycans in cancer. Some overlapped genes were shared by these identified pathways (Figure 2(e)). For example, there were five common genes shared by focal adhesion pathway and Proteoglycans in cancer pathway.

### 3.3. Calculation of MIRA Score to Infer the Regulation Activities of Genes Involved in the Significant Pathways

**Figure 3:**
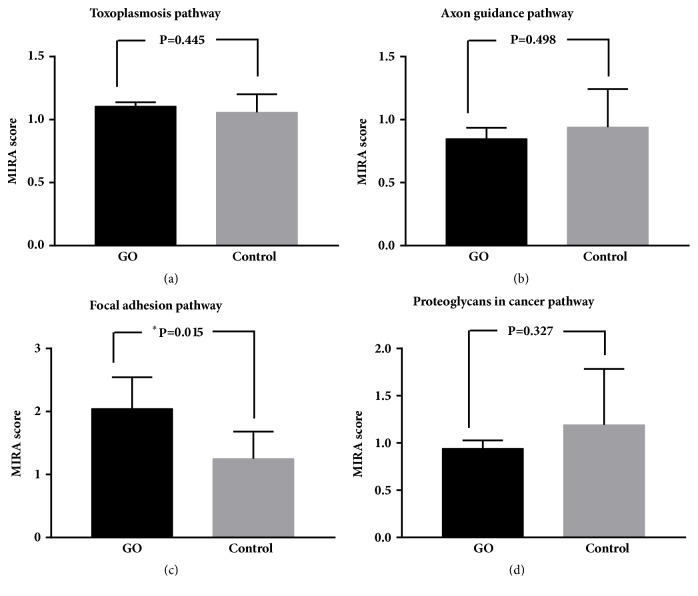
MIRA scores of genes involved in significant pathways between GO patients and normal controls. (a) Toxoplasmosis pathway. (b) Axon guidance pathway. (c) Focal adhesion pathway. (d) Proteoglycans in cancer pathway. MIRA: Methylation-based Inference of Regulatory Activity.

To explore the level of regulatory activity of genes involved in significant pathways, we calculated the MIRA score of each sample. For each significant pathway, we compared MIRA scores between GO patients and normal controls and found that the MIRA score of genes involved in focal adhesion pathway displayed the significant difference between GO and control (p=0.015), which was shown in Figure 3. From Figure 3(c), we observed that MIRA scores of GO patients are greater than normal controls in focal adhesion pathway. This indicates that the regulatory activity of genes in GO patients is more active than controls.

**Figure 4:**
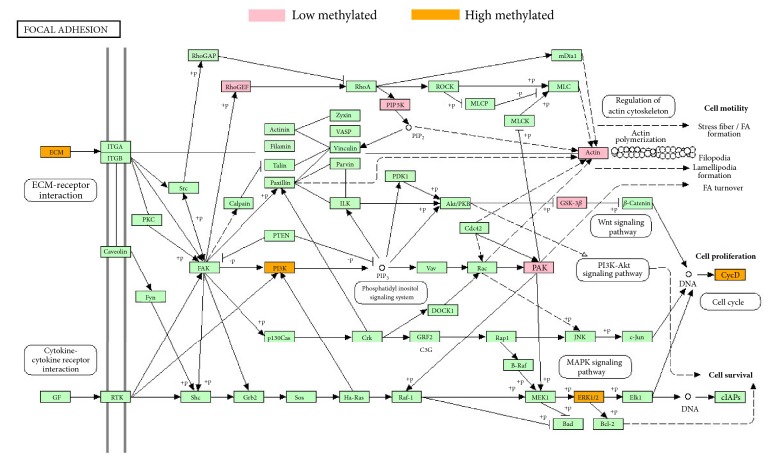
The Focal adhesion pathway in KEGG graph. Genes showing lower methylation level in GO patients were highlighted in pink colors whereas genes showing higher methylation in GO patients were highlighted in orange colors.

Specially, in focal adhesion pathway, we observed 63.6% genes showed lower methylation level in GO patients than in normal controls, which is in accordance with the assumption that the DNA methylation will generally be lower in active regions (Figure 4).

### 3.4. Random Forest Analysis to Extract GO-Related Feature Genes Involved in the Significant Pathways

**Figure 5:**
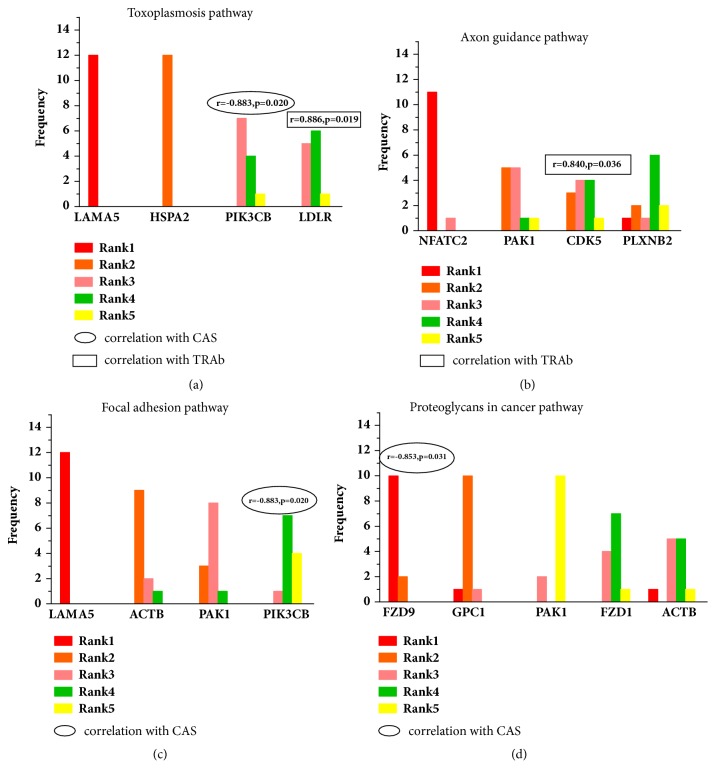
GO-related feature genes extracted by random forest method. In each significant pathway, we extracted those genes whose MDG ranked the top 5 in all of 12 models as GO-related feature genes. (a) Feature genes involved in toxoplasmosis pathway. (b) Feature genes involved in Axon guidance pathway. (c) Feature genes involved in focal adhesion pathway. (d) Feature genes involved in Proteoglycans in cancer pathway.

For each significant pathway, we took the genes involved in pathway as the subset and applied RF method to classify samples and to extract GO-related feature genes. We adopt leave-one-out (LOO) to implement this analysis and to evaluate the classification performance. The classification accuracy rates were 91.7% for toxoplasmosis pathway, 75.0% for Axon guidance pathway, 91.7% for focal adhesion pathway, and 66.7% for Proteoglycans in cancer pathway, respectively. For each significant pathway, we extracted those genes whose MDG ranked the top 5 in all of 12 models as GO-related feature genes. In toxoplasmosis pathway, LAMA5, HSPA2, PIK3CB, and LDLR were extracted. In Axon guidance pathway, NFACT2, PAK1, CDK5, and PLXNB2 were extracted. In focal adhesion pathway, LAMA, ACTB, PAK1, and PIK3CB were extracted. In Proteoglycans in cancer pathway, FZD9, GPC1, PAK1, FZD1, and ACTB were extracted. In addition, PIK3CB (p=0.020) and FZD9 (p=0.031) were found to be associated with CAS; LDLR (p=0.019) and CDK5 (p=0.036) were found to be associated with TRAb (Figure 5).

## 4. Discussions

GO is an orbit autoimmune disease that often occurs in patients with abnormal thyroid function, usually hyperthyroidism. Due to the fact that the pathogenesis mechanism of this disease is not fully understood, the identification of the genes associated with this disease has become an urgent task for clinical treatment. In this study, we performed a genome-scale screen of DNA methylation in peripheral blood samples of Chinese patients and applied pathway analyses to extract potential GO-related feature genes. In previous study, the general enrichment analysis did not find any significant pathway [4]. In present study, the pathway analyses were performed to extract the significant pathways based on the DMRs. Then, for each significant pathway, the MIRA scores were calculated to infer the regulatory activity of genes involved in the pathway. Furthermore, we took the significant pathways as the subsets and applied random forest (RF) method to extract GO-related feature genes. As a result, we identified four significant pathways which might be associated with the occurrence and development of GO. The identified genes involved in the significant pathways, such as LDLR, CDK5, and PIK3CB, were found to be correlated with GO phenotype.

In the practice, some evidences supporting these pathways might be related to GO directly or indirectly. For example, the focal adhesion pathway was found to be significant. The focal adhesion function is both mechanical and responsive. Focal adhesion complex (FAC) is one of the candidate biomarkers of cellular signal transduction pathways. FAC can be involved in regulating inflammatory gene expression via signal transduction pathways such as interleukin 1 (IL-1) signaling [16, 17] or regulating calcium fluxes via phosphatidyl inositol signaling [16, 18], which impacts on inflammatory cascades. FAC has been implicated in the pathogenesis of multiple inflammatory diseases, including inflammatory bowel disease (IBD) and rheumatoid arthritis. It could be used in GO activity assessment and to determine optimal therapeutic strategies in the future. The Proteoglycans in cancer pathway was also significant. As an autoantigen, Proteoglycans can stimulate sequential immune reactions via activation of CD4^+^ T cells, which is observed in autoimmune disease of rheumatoid arthritis [19]. In addition, a recent study reported the latent toxoplasmosis was associated with a mild increase in thyroid hormone production in pregnancy. The observed toxoplasma-associated changes could provide new clues to the complex pathogenesis of autoimmune thyroid diseases [20]. It is also reported that the damage to ocular tissues led to the proposal of phenomena that may be related to pathogenic mechanisms of ocular toxoplasmosis including autoimmune mechanisms [21].

There are some identified genes correlated with GO phenotype involved in the significant pathway. Graves' disease (GD) and GO are typical autoimmune diseases. Meanwhile, oxidative stress (OS) is found to be associated with GD and GO [22]. Once the autoimmune reaction against antigens is initiated, a number of effector mechanisms intervene to cause the pathological alterations of the orbital soft tissue. One of these mechanisms is the release of reactive oxygen species, resulting in OS [23]. It is reported that the level of OS is increased in subjects with GO compared to the other subjects with GD [22]. In this study, we identified a feature gene CDK5 associated with TRAb in Axon guidance pathway. The previous study reported the deregulated CDK5 can promote OS by compromising the cellular antioxidant defense system [24]. The evidence also showed that CDK5 plays an important role in mediating inflammatory [25]. Furthermore, PIK3CB was found to be associated with CAS. It is reported that the activation of PI3-kinase signaling in macrophages, which in turn inhibits NF-*κ*B activation and suppresses proinflammatory gene expression [26]. We also found LDLR was associated with TRAb. Interestingly, a new study found that total and LDL cholesterol correlate with GO activity in patients with a short duration of hyperthyroidism, suggesting a role of cholesterol in the clinical expression of GO. One hypothesis is that the altered inflammatory state of hypercholesterolemia may explain the correlation between GO activity with LDL cholesterol [27].

Although our findings might provide new insights into understand GO disease, it should point out the limitations of the small sample size in the present study. To minimize the impact of sample size on the analysis results, we used strict criterion to select GO patients and their matched controls. By this strict definition, we hope to ensure this explore analysis can have a certain quality. In addition, we chose the statistical methods which are suitable for analyzing high dimensional data with a small sample size. Our future study will enlarge sample size to validate these findings and integrate different data type, including DNA methylation data, gene microarray data, imaging data, and the network information into the prognostic biomarker discovery of GO.

## 5. Conclusion

We performed a Genome-wide DNAm screen in peripheral blood and applied pathway analyses to extract potential GO-related feature genes in Chinese patients with GO. As a result, we identified significant pathways which might be associated with GO and genes involved in the significant pathways, such as LDLR, CDK5, and PIK3CB correlated with GO phenotype. Our study suggested pathway analyses can help understand the potential relationships between the DNA methylation level of some certain genes and their regulation in Chinese GO patients.

## Figures and Tables

**Table 1:** Characteristics of case and control subjects.

	Age	Sex	Height	Weight	Duration	CAS	TRAb
	(year)	(M/F)	(cm)	(cm)	(months)		(U/L)
Case 1	57	M	175	86	6	3	2.14
Case 2	55	M	172	85	1	4	11.39
Case 3	54	M	172	70	3	4	6.92
Case 4	61	M	175	80	2	2	5.13
Case 5	47	M	172	70	6	3	3.37
Case 6	53	M	162	64	11	5	5.3
Control 1	46	M	171	77			<1.75
Control 2	48	M	176	72			<1.75
Control 3	52	M	175	72			<1.75
Control 4	54	M	168	65			<1.75
Control 5	50	M	169	80			<1.75
Control 6	49	M	165	79			<1.75

CAS: Clinical Activity Score.

**Table 2:** The extracted significant pathways.

Pathway Name	KEGG ID	The number of genes involved in the pathway	p-value
Toxoplasmosis	hsa05145	10	<0.001
Axon guidance	hsa04360	10	<0.001
Focal adhesion	hsa04510	11	<0.001
Proteoglycans in cancer	hsa05205	13	0.007

## Data Availability

The data used to support the findings of this study are available from the corresponding author upon request.

## Abbreviations

GO: Graves' Orbitopathy
DNAm: DNA methylation
KEGG: Kyoto Encyclopedia of Genes and Genomes
DMRs: Differentially methylated regions
MAD: Median absolute deviation
CAS: Clinical Activity Score
RRBS: Reduced Representation Bisulfite Sequencing
TSS: Transcription start sites
MIRA: Methylation-based Inference of Regulatory Activity
RF: Random forests
LOO: Leave-one-out
MDG: Mean Decrease Gini
FAC: Focal adhesion complex
IBD: Inflammatory bowel disease
GD: Graves' disease
OS: Oxidative stress.
